# Ultrasensitive Functionalized Polymeric-Nanometal Oxide Sensors for Potentiometric Determination of Ranitidine Hydrochloride

**DOI:** 10.3390/polym14194150

**Published:** 2022-10-03

**Authors:** Eman M. Alshehri, Nawal A. Alarfaj, Salma A. Al-Tamimi, Maha F. El-Tohamy

**Affiliations:** Department of Chemistry, College of Science, King Saud University, P.O. Box 22452, Riyadh 11495, Saudi Arabia

**Keywords:** ranitidine hydrochloride, coated wire sensors, metal oxide nanoparticles, modified potentiometric sensors

## Abstract

Two metal oxide nanoparticles, magnesium oxide nanoparticles (MgONPs) and aluminum oxide nanoparticles (Al_2_O_3_NPs), were synthesized from green sources, Salvia officials and Cuminum cyminum seed extract, respectively. These nanoparticles were used for construction of potentiometric enhancement sensors employed for the estimation of ranitidine hydrochloride (RNT) in authentic powder and commercial products. The electroactive substance ranitidine-phosphotungstate (RNT-PT) was formed by combining RNT with phosphotungstic acid (PTA) in the presence of plasticizing material *o*-nitrophenyloctyl ether (*o*-NPOE). The outcomes showed that the enhanced MgO and Al_2_O_3_ nanosensors behaved linearly across the concentration ranges 1.0 × 10^−9^–1.0 × 10^−2^ and 1.0 × 10^−10^–1.0 × 10^−2^ mol L^−1^, respectively. However, the conventional sensor (RNT-PT) displayed a linearity over 1.0 × 10^−6^–1.0 × 10^−2^ mol L^−1^. Least square equations were calculated as E_mV_ = (54.1 ± 0.5) log (RNT) + 762.33, E_mV_ = (58.6 ± 0.2) log (RNT) + 696.48, and E_mV_ = (52.2 ± 0.7) log (RNT) + 756.76 for enriched nanometal oxides modified and conventional sensors, respectively. The correlation coefficients of regression equations were 0.9997, 0.9995, and 0.9992 for the above suggested sensors, respectively. The recorded results showed excellent sensitivity and selectivity of the modified nanometal oxide sensors for the quantification of the analyzed drug in its authentic samples and commercial products.

## 1. Introduction

Nanoscale materials have recently been identified as a possible key in sensors, material building, biomedical applications and targeting systems for cancer medication. Scientists are still attempting to expand their study field in which the advanced qualities of nanosized substances may be used to modify various sensitive sensors in our modern life [1]. Nanoscale material has a variety of fascinating features that were being studied as workable solutions to a variety of contemporary challenges. It provides a significant new contribution to addressing global and environmental issues [2].

Lately, nanotechnology involves creating intelligent materials with novel features that have just been identified. This is extremely beneficial to analytical chemistry [3,4]. Magnesium oxide (MgO) is a basic oxide with a wide range of uses. It has potential to be used as a caustic adsorbent for harmful chemical waste. MgONPs exhibited excellent optical, electrical, magnetic, thermal, mechanical, and chemical characteristics due to their distinctive architectures and diverse properties. As a result, MgONPs have been widely used in the sectors of catalysis, hazardous waste remediation, and refractory materials [5,6,7].

Aluminum oxide nanoparticles (Al_2_O_3_NPs) is a type of metal oxide nanoparticle used in various biomedical purposes due to their unique physicochemical and structural properties, including resistance to wear, chemicals, and mechanical stresses, as well as good optical properties and a porous large surface area. Another reason for Al_2_O_3_NPs extensive use is their inexpensive preparation cost and ease of handling [8,9].

Pharmaceutical quantitative analysis is required at various stages of medication research and manufacture. As a result, it is advantageous to investigate low-cost, accurate quick techniques that do not involve toxic solvents, sample pretreatment, or extraction processes. Electroanalytical methods have recently become popular for determining medicinal substances. It provides several benefits, such as simplicity of use, fast, power saving, sensitivity, cost-effective, and reliability [10,11].

The most common potentiometric electrodes for detecting medical medications, organic or inorganic chemicals are self-powered electrodes that do not require any other energy sources. These electrodes’ potentiometric measurements are obtained as a result of analyte concentration caused by an electrostatic process that produces an electromotive force (emf) between the surface of the indicator sensor and the reference one [12]. Recently, the scientists exhibited an interest in the engineering of new molecular carriers which possess excellent electrical conductivity, lipophilic property, capability of reversibility of binding of metal ions, and selectivity, and permitting their permeation via the sensor membrane over the other ions [13,14,15,16,17].

The synthesized MgONPs and Al_2_O_3_NPs have structures and sizes, allowing them to be used to build a variety of catalytic sensing systems with low detection limits, large concentration ranges, increased percentage of recoveries, good repeatability, and operation at room temperature [18,19].

**Figure 1:**
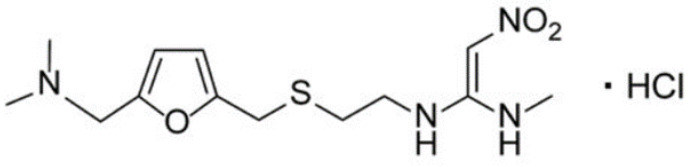
Structural formula of ranitidine hydrochloride.

Ranitidine hydrochloride (RNT, Figure 1) are H2 antagonists and histamine which are competitively inhibited at the H2 receptor in parietal cells by H2 antagonists. They prevent both meal-stimulated acid release and the regular acid secretion by parietal cells. They accomplish this by using two pathways: When the H2 receptors are blocked, histamine released by enterochromaffin-like (ECL) cells in the stomach is prevented from binding to parietal cells’ H2 receptors, which stimulate acid secretion. Other substances that stimulate acid secretion (such as gastrin and acetylcholine) also have less of an effect on parietal cells [20,21].

The use of modified metal oxide sensors to determine and quantify dose form pharmaceuticals is now gaining a lot of interest. The goal of this research is to create modified coated wire sensors enhanced with MgONPs and Al_2_O_3_NPs that are very sensitive and selective. The results show that the created electrodes have several advantages, including simplicity, versatility, and cost-effectiveness.

## 2. Materials and Methods

### 2.1. Chemicals

Ranitidine hydrochloride (RNT-HCl), magnesium nitrate hexahydrate, aluminum nitrate hexahydrate, hydrochloric acid 37%, sodium hydroxide, acetone 99.9%, tetrahydrofuran (THF) 97.0%, methanol 99.9%, ortho-nitrophenyloctyl ether (*o*-NPOE), phosphomolybdic acid, ethanol 99.9%, and polyvinyl chloride (PVC, high molecular weight) were provided by Sigma Aldrich (Hamburg, Germany). The medication form of RNT HCl, Ranimax^®^ tablets (150 mg ranitidine hydrochloride/tablet) were obtained from Jazeera Pharmaceutical Industries (Riyadh, Saudi Arabia).

### 2.2. Instrumentation

All experimental studies were carried out using a 211-HANNA pH meter (HANNA instruments, Smithfield, VA, USA). Another pH meter (Metrohm-744) was used to adjust the pH of the analytical samples. The characterization of the synthesized nanomaterials was performed using spectroscopic devices including, Shimadzu-spectrophotometer (Shimadzu Corporation, Kyoto, Japan), BX-spectrometer (PerkinElmer, Waltham, MA, USA). The surface structure of the formed nanomaterials was examined under scanning electron microscopes (SEM, JSM-7610F JEOL, Tokyo, Japan). The size of the metal oxide nanoparticles was evaluated from the spectra of 6000-X-ray diffractometer (XRD, Shimadzu, Kyoto, Japan). To confirm the presence of Mg and Al metals, Energy-Dispersive X-Ray Spectroscopy (EDX) analysis was performed using a SEM microscope connected with EDX.

### 2.3. Green Preparation of Nanoparticles

**Figure 2:**
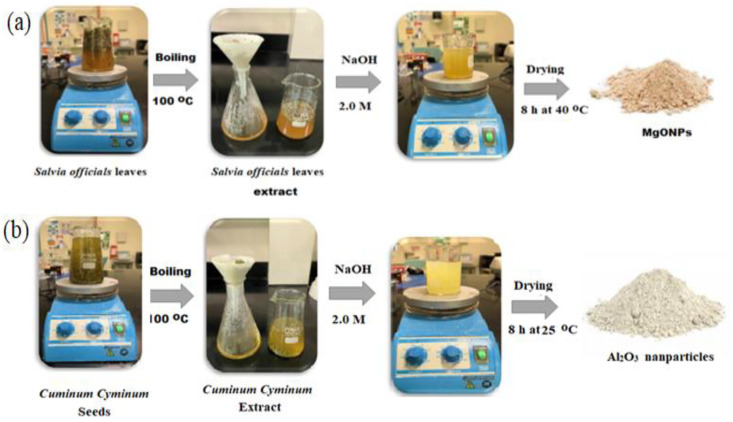
Green synthesis of (**a**) MgONPs using *Salvia officials* leaves extract and (**b**) Al_2_O_3_NPs using *Cuminum cyminum* seeds extract.

The synthesis of MgONPs starts by preparing *Salvia officials* extract solution; 5.0 g of dried *Salvia officials* leaves were added to 500 mL deionized water and boiled for an hour at 100 °C. The formed extract was filtered using a filter paper (Whatman No. 1). The prepared extract was used for the synthesis of MgONPs. Approximately, 10 mL of *Salvia officials* extract was mixed with 50 mL of freshly prepared 0.1 mol L^−1^ of magnesium nitrate hexahydrate solution under magnetic agitation at 80 °C for 2 h. The pH was adjusted in the range of 10–12 using 2.0 mol L^−1^ sodium hydroxide solution. The MgONPs precipitate was collected after centrifugation of the solution for 10 min at 10,000 rpm. To remove the excess of Mg (NO_3_)_2_ and plant material from the MgONPs precipitate, the collected precipitate was washed three times with absolute ethanol, then oven-dried at 40 °C for 8 h. The resulting material was then finely powdered with mortar and pestle and then calcined in a muffle furnace at 450 °C for 30 min (Figure 2a) [22].

For Al_2_O_3_NPs synthesis, 10 g of *Cuminum cyminum* seed was stirred in 500 mL of deionized water and boiled for 2 h at 100 °C. The formed extract was then filtered using Whatman filter paper No. 1. The clear cumin seed extract was obtained. To a 50 mL aqueous solution of 0.1 mol L^−1^ Al (NO_3_)_3_, 5 mL *Cuminum cyminum* seed extract was added; 2.0 mol L^−1^ NaOH was used to adjust the pH of the resulting solution to pH 8. A change in the color of the solution to brown indicated the production of Al_2_O_3_NPs. At normal temperature (25 °C), the reduction of aluminum ions to nano aluminum takes 2 h. The synthesized nanoparticle solution was centrifuged at 10,000 rpm for 10 min prior to being distributed in deionized water and left to dry (Figure 2b) [23].

### 2.4. Spectroscopic and Microscopic Characterization of Nanoparticles

The pre-synthesized metal oxide nanoparticles were subjected to different spectroscopic and microscopic techniques to confirm their nanoscale features. The optical properties of MgONPs and Al_2_O_3_NPs were measured using UV-Vis spectrophotometer in the range of 200 to 800 nm. To confirm the possible functional groups, present on the surface of the synthesized MgONPs and Al_2_O_3_NPs, FT-IR spectrophotometer was used in the range from 400–4000 cm^−1^. The size estimation of the formed metal oxide nanoparticles was calculated from the recorded XRD peaks measured at a voltage of 40 kV and a current of 40 mA with Cu Ka radiation of wavelength 0.15406 nm. The morphology of the nanoparticles was studied using SEM and the elemental composition of the formed nanomaterials was determined using an EDX spectrometer.

### 2.5. Preparation of Standard Solution

A preparation of standard solution of 1.0 × 10^−2^ mol L^−1^ was conducted by dissolving 0.35 g of RNT in 100 mL of distilled water. Analytical solutions with different concentrations were obtained from the dilution of the standard one to the desired concentrations using distilled water.

### 2.6. Formation of Ion-Pair Complex

By mixing 50 mL of 1.0 × 10^−2^ mol L^−1^ RNT solution with the equivalent volume of 1.0 × 10^−2^ mol L^−1^ PTA solution, ranitidine hydrochloride-phosphotungstate (RNT-PT) was obtained. RNT-PT was produced as a faint yellow precipitate. The produced precipitate was dried overnight at room temperature after being filtered and washed.

### 2.7. Sensor Design and Membrane Composition

**Figure 3:**
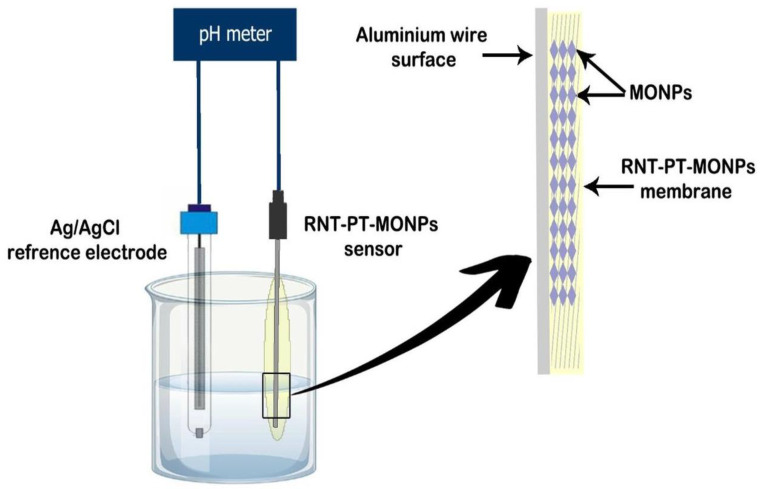
Illustration of modified RNT-PT-MONPs and the potentiometric sensor system.

RNT-PT, RNT-PT, RNT-PT-MgONPs, and RNT-PT-Al_2_O_3_NPs were designed by suspending PVC (190 mg), electroactive complex (RNT-PT, 10 mg), and plasticizer (*o*-NPOE, 0.35 mL) in 5 mL of organic solvent (THF). The blended solution was allowed to evaporate in a Petri dish with a diameter of 3 cm until an oily membrane solution developed. To create the conventional RNT-PT sensor, an Al wire that had been polished and cleaned with acetone was dipped into the membrane mixture numerous times. A plastic membrane mixture including MgONPs or Al_2_O_3_NPs (5 g), PVC (190 mg), RNT-PT-MgONPs or RNT-PT-Al_2_O_3_NPs ion pair (10 mg), and *o*-NPOE plasticizer (0.35 mL) in 5 mL of THF were performed in order to create the modified sensors. At room temperature, a well-homogeneous distributed membrane mixture was created by swirling constantly for 15 min. A thin layer on the surface of the sensors was simulated using the generated membrane mixture. After drying, sensors were repeatedly submerged in the coated membrane solution to create a thickly coated wire membrane (Figure 3).

### 2.8. Calibration Graphs

The designed sensors and Ag/AgCl as a reference electrode were used to assess and evaluate 25 mL of 1.0 × 10^−10^–1.0 × 10^−2^ mol L^−1^ RNT standard solution, separately. The calibration graphs of each sensor displayed the potential values as a function of -logarithm RNT concentrations.

### 2.9. Optimization of Potential Readings’ Conditions

To investigate the pH impact of 1.0 × 10^−3^ mol L^-−1^ of the analyte RNT solution on the potential value of the designed sensors, it was used in conjunction with a reference sensor Ag/AgCl and a combined glass electrode, and the pH was measured in 50 mL aliquots of the tested drug; 0.1 mol L^−1^ HCl or a few drops of 0.1 mol L^−1^ NaOH were used to modify the pH. The pH values as a function of potential measurements of each sample were shown on pH graphs. The standard approach for evaluating the selectivity of the fabricated sensor in such studies is the separate solution method [24]. The dynamic response time was calculated using the potential response for drugs with varying concentrations from 1.0 × 10^−9^–1.0 × 10^−2^ mol L^−1^.

### 2.10. Analytical Applications

Five Ranimax^®^ tablets (150 mg/tablet) were finely crushed, and 0.53 g was dissolved in distilled water to make a standard solution of 1.0 × 10^−2^ mol L^−1^. To achieve varied concentrations of RNT in the range of 1.0 × 10^−6^–1.0 × 10^−3^ mol L^−1^, working solutions were created. To determine each concentration of the tested medication, the recommended sensors RNT-PT-MgONPs and RNT-PT-Al_2_O_3_NPs were utilized individually.

## 3. Results and Discussion

### 3.1. Characterization of the Synthesized Nanoparticles

**Figure 4:**
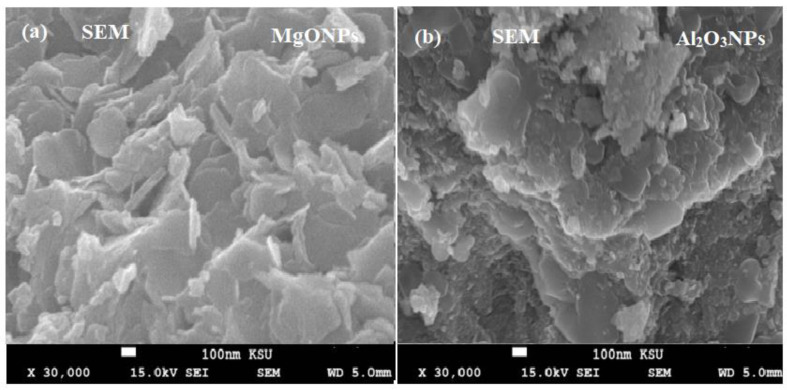
Scanning electron microscope images of (**a**) MgONPs and (**b**) Al_2_O_3_NPs.

Microscopic methods such as SEM were used to characterize the synthesized MgONPs and Al_2_O_3_NPs. Using field emission scanning electron microscopy (FESEM) showed the morphology and shape of the produced MgONPs and Al_2_O_3_NPs at 30,000× magnification. The micrographs of MgONPs and Al_2_O_3_NPs have been displayed in Figure 4a,b. They showed the widely distributed hexagonal particles, but they are all connected or closely related. The size of the produced particles ranges from a few nanometers to 100 nm. The smaller size particles are crammed together so tightly that they appeared to be embedded in the surface. The synthesized samples did not contain any additional structures or morphologies other than MgONPs and Al_2_O_3_NPs.

**Figure 5:**
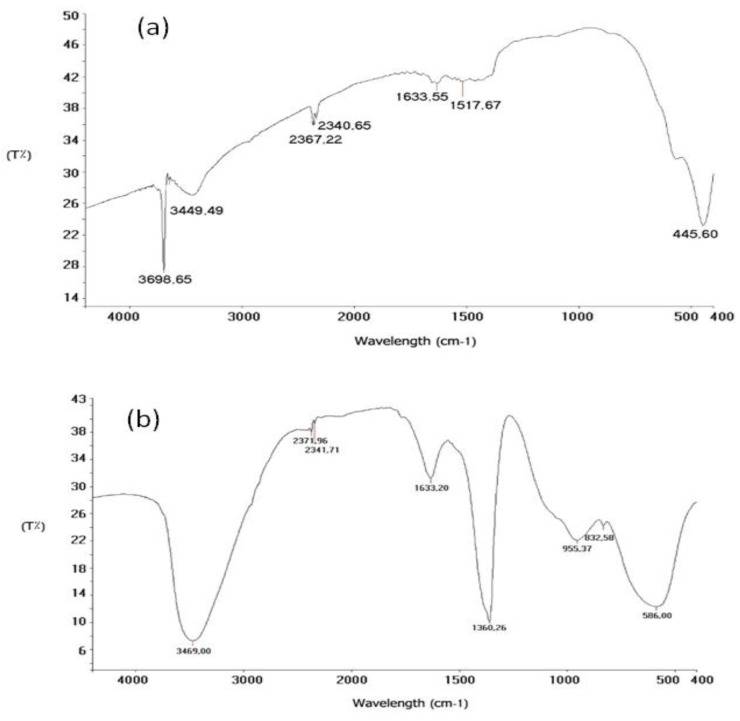
FT-IR spectra of the green synthesized (**a**) MgONPs and (**b**) Al_2_O_3_NPs using *Salvia officials* leaves and *Cuminum cyminum* seed extract, respectively, at a wavenumber range from 4000 to 400 cm^−1^.

Furthermore, FT-IR analysis was applied to confirm various functional groups that present in the formed metal oxide nanoparticles. Notable remarkable bands at 3698.65 cm^−1^ and 3449.49 cm^−1^ in the FT-IR spectra of the synthesized MgONPs were attributed to be associated to OH-stretching groups. A significant band at 2367.22 cm^−1^ verified the existence of CH-stretching. The bands at 1517 cm^−1^ and 1488 cm^−1^ and 1378 cm^−1^ were found to be associated to carboxylic acid C=O groups and equines C=C groups, respectively. The peak at 445.60 cm^−1^ (Figure 5a) verified the synthesis of MgO metal stretching. For OH-stretching groups in Al_2_O_3_NPs, well-defined bands were found at 3798 cm^−1^, 3693.25 cm^−1^, and 3496 cm^−1^. The presence of CH-stretching was established by the absorption band 2341.71 cm^−1^. The band at 1633.20 cm^−1^ was determined to be associated to a carboxylic acid’s C=O group (Figure 5b).

**Figure 6:**
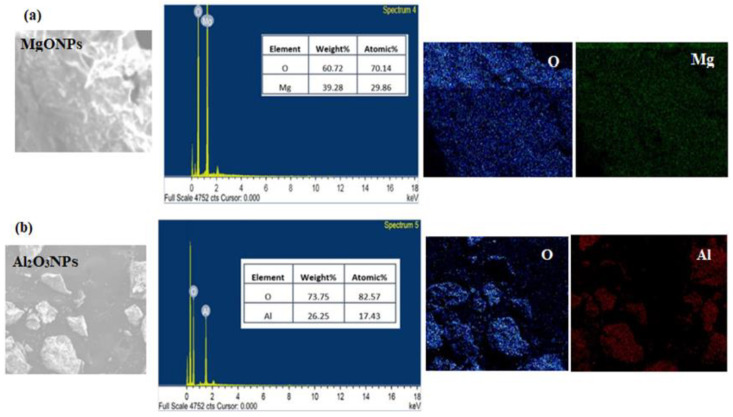
EDX analysis and elemental X-ray mapping of green synthesized (**a**) MgONPs and (**b**) Al_2_O_3_NPs using *Salvia officials* leaves and *Cuminum cyminum* seed extract, respectively.

The presence of magnesium and aluminum elements in MgONPs and Al_2_O_3_NPs was discovered utilizing a SEM equipped with an EDX spectrometer to examine their EDX profiles. The content percentages of Mg and Al nanoparticles were 39.28% Mg and 60.72% O for MgONPs (Figure 6a), and 26.25% AL and 73.75% O for Al_2_O_3_NPs (Figure 6b), according to the recorded profiles. The maximum intensity peaks for Mg and AL were 0.8 keV and 0.8 keV, respectively.

**Figure 7:**
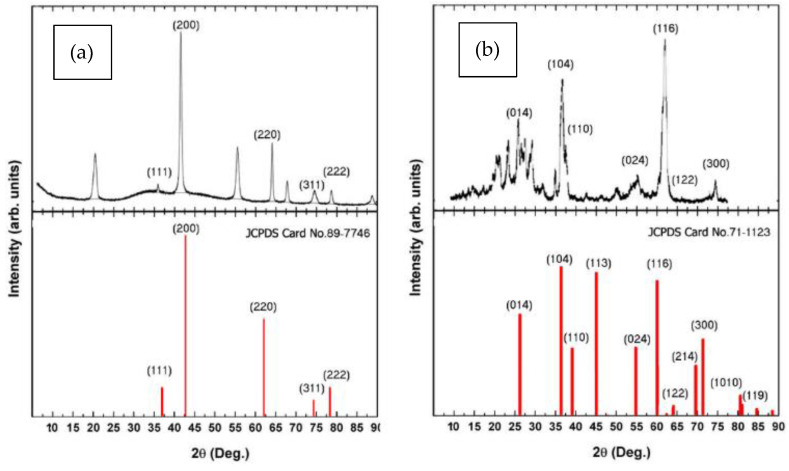
XRD patterns of green synthesized (**a**) MgONPs and (**b**) Al_2_O_3_NPs using *Salvia officials* leaves and *Cuminum cyminum* seed extract, respectively, at Cu-kα and (k = 1.54060 Å).

XRD is an analytical technique for measuring and quantifying various crystalline structures in the samples under investigation (Figure 7a). This analysis was carried out utilizing a Cu-kα XRD diffractometer at (k = 1.5406 Ǻ). XRD patterns of MgONPs and their exhibited distinct peaks at 2θ = 37.8°, 46.6°, 63.8°, 74.3, and 78.9 corresponding to MgO of (1 1 1), (2 0 0), (2 2 0), (3 1 1), and (2 2 2), respectively. These values can be assigned as a high hexagonal crystalline phase, and these results were matched to the JCPDS file of MgO (No. 89-7746).

For Al_2_O_3_NPs, XRD investigation (Figure 7b) revealed a series of diffraction peaks at 2θ = 26.8°, 37.62°, 39.63°, 55.95°, 60.2°, 64.8°, and 72.5°, which correspond to the crystal planes of (0 1 4), (1 0 4), (1 1 0), (0 2 4), (1 1 6), (1 2 2), and (6 2 0). All of the diffraction peaks matched a pure cubic structure of Al_2_O_3_ (JCPDS Card no. 71-1123).

### 3.2. The Fabricated Sensors Behavior

RNT combines with PT to generate a stable RNT-PT ion pair that can be dissolved in organic solvents such as THF. In the presence of PVC, the solvent mediator *o*-NPOE was applied with the RNT-PT ion pair. The critical response characteristics of the fabricated sensors were determined and presented in Table 1.

**Figure 8:**
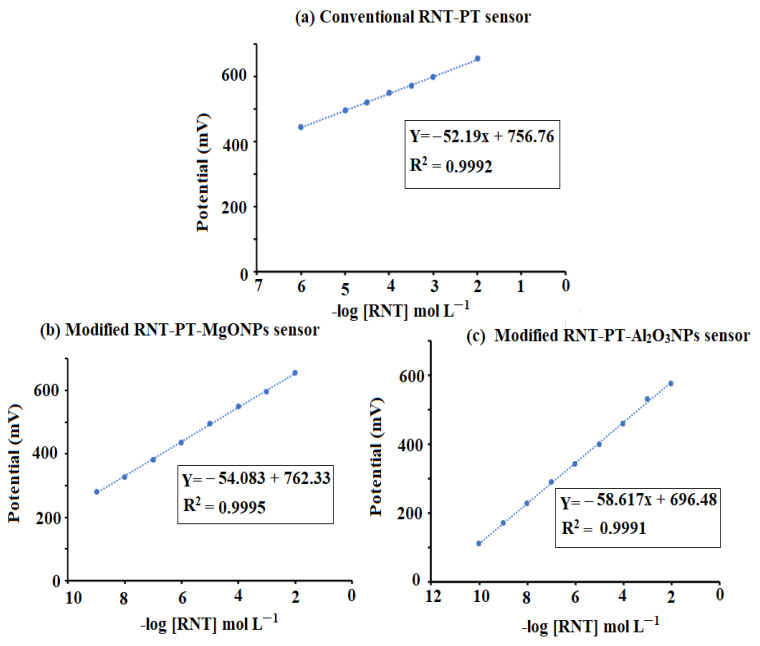
Calibration graphs of the fabricated (**a**) conventional RNT-PT, (**b**) modified RNT-PT-MgONPs, and (**c**) modified RNT-PT-Al_2_O_3_NPs sensors.

The fabricated sensors gave Nernstian responses with slopes of 52.2 ± 0.7, 54.1 ± 0.5, and 58.6 ± 0.2 mV over the drug concentration ranges of 1.0 × 10^−6^–1.0 × 10^−2^, 1.0 × 10^−9^–1.0 × 10^−2^, and 1.0 × 10^−10^–1.0 × 10^−2^ mol L^−1^ for conventional RNT-PT and modified RNT-PT-MgONPs and RNT-PT-Al_2_O_3_NPs, respectively (Figure 8a,c).

The presence of MgONPs and Al_2_O_3_NPs nanocrystals, which enhanced the membrane sensitivity of the fabricated modified sensors and their stability during the potentiometric measurements, is responsible for the excellent performance of the potentiometric RNT sensors. The high sensitivity of the modified sensors using Al_2_O_3_NPs more than that modified by MgONPs was due to the high dielectric constant of Al_2_O_3_NPs (ε= 8.4) than MgONPs (ε= 3.2) which enhanced the conductivity of the sensor transducer surface and increased the detection of the target analyte. Furthermore, metal oxides’ outstanding electrical and extraordinary capacity features, such as high charge transfer at nanomaterial interfaces, are essential when nanomaterials are exploited as transducing materials in sensing applications [25].

**Figure 9:**
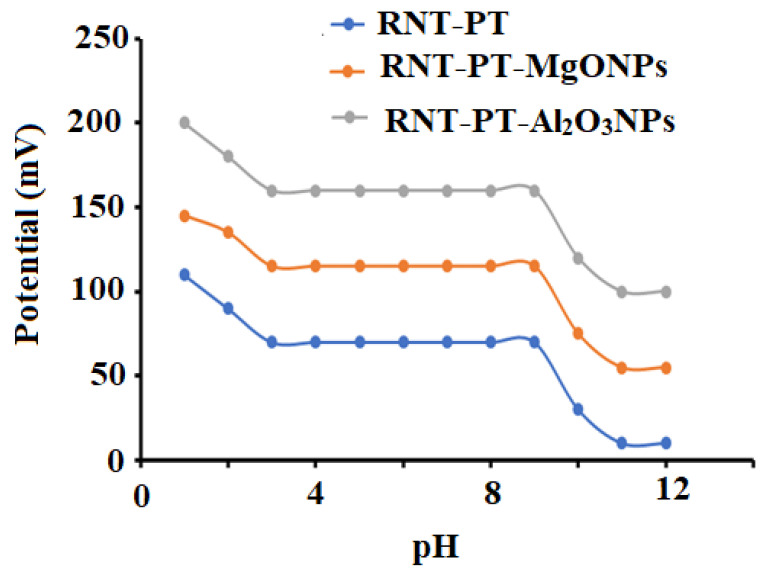
Influence of pH on the suggested conventional RNT-PT and modified metal oxide RNT-PT-MgONPs and RNT-PT-Al_2_O_3_NPs coated wire sensors using 1.0 × 10^−3^ mol L^−1^ of RNT solution.

The effect of pH on the potential of conventional and modified sensors was investigated in order to determine the safe pH range for RNT detection. The results showed that in the pH range 3–9, conventional RNT-PT, modified RNT-PT-MgONPs, and RNT-PT Al_2_O_3_NPs sensors were essentially independent, and this range can be used to determine RNT in a safe manner (Figure 9).

To determine the selectivity of the developed sensors for the drug under investigation, the proposed sensors were evaluated to analyze 1.0 × 10^−3^ mol L^−1^ of different inorganic cations, sugars, amino acids, and related RNT chemical structure compound. The modified sensors RNT-PT-MgONPs and RNT-PT-Al_2_O_3_NPs showed excellent selectivity. The construction of the sensors became more conductive and therefore more selective for the medication under study due to the inclusion of metal oxide nanoparticles with significant surface area and physicochemical characteristics. The RNT^+^ ions’ free energy transfer between the membrane and the surrounding medium may be the cause of this selectivity. The free energy of RNT^+^ transfer between the aqueous and coated membrane phases was often referred to as the selectivity of RNT coated membrane sensors. Due to the ionic size differences, mobility, and permeability of the proposed sensors, compared to RNT^+^, no interference was observed in the detection of inorganic cations (Table 2).

It is not surprising that the addition of metal oxide nanoparticles in the membrane composition or the introduction of a thin layer as an intermediate layer improved the stability, sensitivity, and selectivity of the modified sensors. The metal oxides (MgONPs and Al_2_O_3_NPs) belong to the nanostructures elements that gained excellent physical and chemical features that bulk their counterparts. The high surface area-to-volume ratio and semiconducting characteristics recognized them. The electroactive surface area is increased, and electron transport between the sensor membrane and the ions of analytes is improved [26].

### 3.3. Quantification of Ranitidine Hydrochloride

The designed sensors were used to quantify RNT in its bulk form and the percentage recoveries were 99.35 ± 0.5, 99.71 ± 0.3 % and 99.38 ± 0.2, and 99.78 ± 0.4 using RNT-PT, RNT-PT-MgONPs, and RNT-PT-Al_2_O_3_NPs sensors, respectively (Table 3). The advanced properties of the additional nanoparticles were linked to the upgraded sensors’ sensitivity. Furthermore, it was found that the Al_2_O_3_NPs-modified sensor had high sensitivity and selectivity for the examined drug, owing to Al_2_O_3_NPs having a higher dielectric constant than MgONPs.

### 3.4. Method Validation

According to ICH requirements, the suggested analytical procedure was guaranteed and validated [27]. The developed RNT-PT-MgONPs and RNT-PT-Al_2_O_3_NPs sensors showed a wide range of linear concentration correlations over 1.0 × 10^−9^–1.0 × 10^−2^, 1.0 × 10^−10^–1.0 × 10^−2^ mol L^−1^, respectively, with respect to 1.0 × 10^−6^–1.0 × 10^−2^ mol L^−1^ for the conventional coated wire type. The regression equations were E_mV_ = (54.1 ± 0.5) log (RNT) + 762.33, E_mV_ = (58.6 ± 0.2) log (RNT) + 696.48 for enhanced nanometal oxides, respectively. The conventional type RNT-PT showed a potential response of E_mV_ = (52.2 ± 0.7) log (RNT) + 756.76 with correlation coefficients 0.9997, 0.9995, and 0.9996 for the RNT-PT-MgONPs, RNT-PT-Al_2_O_3_NPs, and RNT-PT sensors, respectively.

The potential readings of the developed sensors were obtained after each sensor slope was reduced by 17.9 mV to determine the lower limit of detection (LOD). The obtained results were found to be 4.9 × 10^−10^, 5.0 × 10^−11^, and 5.0 × 10^−7^ mol L^−1^ for the above described sensors, respectively. The devised potentiometric method was tested on nine samples, and the (mean SD) values for the aforementioned sensors were 99.040.7%, 99.490.4%, and 99.540.5%, respectively (Table 3). Through inter-day and intra-day assays, the intermediate precision was also assessed, and the percentage relative standard deviation (%RSD) was computed. The results showed that the %RSD for the manufactured RNT-PT-MgONPs and RNT-PT-Al_2_O_3_NPs were 0.6% and 0.6%, respectively, during intra-day and inter-day. All values fall below the advised value (2.0%), suggesting high precision (Table 4). By adding an acetate buffer with a pH of 50.5, the robustness of the suggested probe was assessed, and the percentage recoveries for RNT-PT, RNT-PT-MgONPs, and other compounds were found to be 99.10.8, 99.500.6, and 99.54 0.2%, respectively. A second investigation was conducted using a different pH meter model to confirm the robustness of the suggested technique (Jenway-3510). For the tested sensors, the computed mean percentage recoveries were 99.2 0.7%, 99.49 0.4%, and 99.5 0.3%. The results showed that the proposed method’s data were accepted in conjunction with other data, and no discernible differences were found.

### 3.5. Quantification of the Drug in Its Tablets

In order to determine the analytical applicability of the sensors that have been developed, RNT was detected in its pharmaceutical form (Ranimax^®^ 150 mg/tablet). The recorded readings were measured versus different concentrations of RNT samples, and the percentage of recoveries was evaluated.

The outcomes were 99.00 ± 0.26, 99.40 ± 0.37, and 99.45 ± 0.50 for the above-mentioned sensors, respectively. It was discovered that the modified sensor RNT-PT-Al_2_O_3_NPs was more sensitive to RNT determination than RNT-PT-MgONPs sensor. The greater dielectric constant of Al_2_O_3_ over MgO may explain how the conductivity of RNT-PT-Al_2_O_3_NPs is higher than that of RNT-PT-MgONPs. The results obtained were statistically analyzed using t-student’s test and F-test [28]. The results were compared with those achieved by the potentiometric method [29], which is established at the formation of ICPE electrode using sodium tetraphenyl borate. The results showed that the suggested sensors have a high sensitivity for detecting RNT in dosage forms (Table 5).

An analytical comparison was performed in Table 6 to show the advanced sensitivity and efficiency of the suggested potentiometric modified sensors for the determination of RNT with other previously reported analytical techniques [30,31,32,33,34,35,36,37,38,39]. The comparative results showed that the suggested modified sensors using metal oxide nanoparticles exhibited higher sensitivity over a wide concentration range than those reported by the previously developed sensors.

## 4. Conclusions

Two coated wire membrane sensors modified with magnesium oxide and aluminum oxide nanoparticles were used in the proposed potentiometric investigation for ranitidine hydrochloride detection. The modified sensors’ potential readings were compared to those of a conventional sensor. Due to their sensitivity and selectivity, the created sensors proved to be superior to other traditional sensors. Furthermore, the employment of metal oxide nanoparticles as coated membrane modifiers resulted in good selectivity in measuring the chosen medication, with a wide linear concentration range and low limit of detection. Consequently, the metal oxide enriched membrane sensors can be utilized for regular ranitidine hydrochloride analysis in pharmaceutical companies, hospitals, and research labs.

## Figures and Tables

**Table 1:** Electrochemical response characteristics of conventional coated wire RNT-PT, modified RNT-PT-MgONPs, and RNT-PT-Al_2_O_3_NPs sensors.

Parameters	Conventional RNT-PT Sensor	Modified RNT-PT-MgONPs Sensor	Modified RNT-PT-Al_2_O_3_NPs Sensor
Slope (mV. Decade^−1^)	52.2 ± 0.7	54.1 ± 0.5	58.6 ± 0.2
Intercept	756.76	762.33	696.48
Correlation coefficient, r	0.9992	0.9997	0.9995
Linear range (mol L^−1^)	1.0 × 10^−6^–1.0 × 10^−2^	1.0 × 10^−9^–1.0 × 10^−2^	1.0 × 10^−10^–1.0 × 10^−2^
LOD	5.0 × 10^−7^	4.9 × 10^−10^	5.0 × 10^−11^
Response time/s	50	30	25
Working pH range	3–9	3–9	3–9
Lifetime/day	20	40	50
Temperature, °C	25	25	25
Accuracy (%)	99.04 ± 0.73	99.49 ± 0.40	99.54 ± 0.53

**Table 2:** Selectivity coefficient (K_Pot_ RNT^+^) of conventional coated wire RNT-PT and modified RNT-PT-MgONPs and RNT-PT-Al_2_O_3_NPs sensors using the separate solution method using 1.0 × 10^−3^ mol L^−1^ RNT.

Interfering Species	Conventional RNT-PT Sensor	Modified RNT-PT-MgONPs Sensor	Modified RNT-PT-Al_2_O_3_NPs Sensor
Fe^3+^	3.6 × 10^−3^	1.4 × 10^−4^	4.9 × 10^−4^
Ca^2+^	7.5 × 10^−3^	1.6 × 10^−5^	7.7 × 10^−5^
Cr^3+^	4.1 × 10^−3^	5.2 × 10^−5^	5.4 × 10^−4^
K^+^	5.5 × 10^−3^	5.8 × 10^−4^	4.6 × 10^−4^
N^a+^	6.9 × 10^−3^	4.2 × 10^−4^	3.5 × 10^−5^
Ag^+^	2.1 × 10^−3^	6.8 × 10^−5^	1.6 × 10^−4^
Mg^2+^	6.2 × 10^−3^	8.4 × 10^−5^	8.1 × 10^−4^
Lactose	5.2 × 10^−3^	6.3 × 10^−4^	2.4 × 10^−5^
Glycine	8.5 × 10^−3^	4.5 × 10^−5^	1.5 × 10^−4^
Histidine	6.3 × 10^−3^	3.6 × 10^−4^	2.3 × 10^−5^
Leucine	7.9 × 10^−3^	9.8 × 10^−4^	3.9 × 10^−4^
Niperotidine	9.5 × 10^−3^	3.5 × 10^−4^	4.8 × 10^−4^

**Table 3:** Statistical analysis of data obtained from the determination of RNT in bulk powder using conventional coated wire RNT-PT, RNT-PT-MgONPs, and RNT-PT-Al_2_O_3_NPs sensors.

	Conventional RNT-PT Coated Wire Sensor			Modified RNT-PT MgONPs Sensor			Modified RNT-PT-Al_2_O_3_NPs Sensor		
	Taken −log (RNT) mol L^−1^	Found mol L^−1^	% Recovery	Taken −log (RNT) mol L^−1^	Found mol L^−1^	% Recovery	Taken −log (RNT) mol L^−1^	Found mol L^−1^	% Recovery
Statistical Analysis	6.00	5.96	99.33	9.00	9.00	100.00	9.00	8.98	99.78
	5.30	5.23	98.68	9.50	9.45	99.47	8.50	8.50	100.00
	5.00	5.00	100.00	8.00	7.97	99.63	8.00	7.95	99.38
	4.30	4.24	98.60	7.50	7.45	99.33	7.00	7.00	100.00
	4.00	3.95	98.75	7.00	6.98	99.71	6.50	6.45	99.23
	3.30	3.28	99.39	5.50	5.50	100.00	6.00	5.99	99.83
	3.00	3.00	100.00	4.00	3.91	98.75	4.00	4.00	100.00
	2.30	2.28	99.13	2.50	2.49	99.60	3.50	3.44	98.29
	2.00	1.95	97.50	2.00	1.98	99.00	3.00	2.98	99.33
Mean ± SD	99.04 ± 0.73			99.49 ± 0.40			99.54 ± 0.53		
n	9			9			9		
Variance	0.54			0.16			0.28		
* %SE	0.24			0.13			0.18		
%RSD	0.74			0.40			0.53		

* SE (%Error) = %RSD/√n.

**Table 4:** Intra- and inter-day assay of ranitidine hydrochloride by using the modified RNT-PT-MgONPs and RNT-PT-Al_2_O_3_NPs coated wire sensors.

Precision Test		Taken −log (RNT) mol L^−1^	% Recovery ^a^	% RSD ^b^	% Error ^c^
RNT-PT-MgONPs	Intra-day precision	10.00	99.50 ± 0.5	0.5	0.32
		5.00	98.83 ± 1.2	1.2	0.70
		2.00	99.83 ± 0.2	0.2	0.12
	Inter-day precision	10.00	98.53 ± 1.2	1.2	0.72
		5.00	98.83 ± 0.8	0.8	0.50
		2.00	97.67 ± 0.6	0.6	0.36
RNT-PT-Al_2_O_3_NPs	Intra-day precision	9.00	99.63 ± 0.3	0.3	0.17
		6.00	99.23 ± 0.7	0.7	0.40
		3.00	98.37 ± 0.8	0.8	0.45
	Inter-day precision	9.00	99.11 ± 0.4	0.4	0.24
		6.00	98.33 ± 1.3	1.3	0.73
		3.00	98.79 ± 0.6	0.6	0.33

^a^ Mean of three determinations, ^b^ %RSD = (SD/Mean) ×100, ^c^ % Error = %RSD/√n.

**Table 5:** Statistical analysis of data obtained from the determination of RNT in Ranimax^®^ tablets, 150 mg/tablet, using conventional coated wire RNT-PT and modified RNT-PT-MgONPs and RNT-PT-Al_2_O_3_NPs sensors.

	Conventional RNT-PT Coated Wire Sensor			Modified RNT-PT MgONPs Sensor			Modified RNT-PT-Al_2_O_3_NPs Sensor		
	Taken −log (RNT) mol L^−1^	Found mol L^−1^	% Recovery	Taken −log (RNT) mol L^−1^	Found mol L^−1^	% Recovery	Taken −log (RNT) mol L^−1^	Found mol L^−1^	% Recovery
Statistical Analysis	6.00	5.94	99.0	9.0	8.98	99.8	7.0	6.98	99.7
	5.00	4.97	99.4	8.0	7.97	99.6	6.0	5.99	99.8
	4.30	4.26	99.1	7.0	6.96	99.3	5.0	4.95	99.0
	4.00	3.96	99.0	6.0	5.97	99.5	4.0	4.00	100.0
	3.00	2.97	99.0	3.0	2.99	98.7	3.0	2.96	98.7
	2.00	1.97	98.5	2.0	1.99	99.5	2.0	1.99	99.5
Mean ± SD	99.00 ± 0.26			99.40 ± 0.37			99.45 ± 0.50		
n	6			6			9		
Variance	0.07			0.14			0.25		
%SE *	0.11			0.15			0.20		
%RSD	0.26			0.33			0.50		
*t*-test	0.569 (2.228) **			1.08 (2.228) **			1.13 (2.228) **		
F-test	2.85 (5.05) **			1.43 (5.05) **			1.25 (5.05) **		
Reported method [27]	99.13 ± 0.45								
	6								
	0.25								
	0.07								

* SE (%Error) = %RSD/√n, ** The tabulated values of ‘‘t’’ and ‘‘F’’ at confidence level *p* = 0.05 [28].

**Table 6:** A comparative study between the results obtained from the determination of RNT using the Potentiometric method by using modified RNT-PT-MgONPs and RNT-PT-Al_2_O_3_NPs sensors, and the previously reported analytical techniques.

Analytical Method	Reagent	Linearity	LOD	Reference
Spectrophotometry	RNT, ninhydrin	8.98 × 10^3^–9.90 × 10^4^ µg L^−1^	0.0997 µg mL^−1^	[30]
Chemiluminescence	RNT, S, N co-doped carbon quantum dots	0.5–50 μg mL^−1^	0.12 µg mL^−1^	[31]
Fluorescence	RNT, CdS quantum dots	0.50–15.0 μg mL^−1^	0.38 µg mL^−1^	[32]
Chromatography	RNT, RP-HPLC method, ortho-phosphoric acid 0.1% and acetonitrile pH 3.5 (25:75, %v/v)	5–25 μg mL^−1^	1.35 µg mL^−1^	[33]
Electrochemical	RNT, poly(dopamine) modified carbon paste electrode	1.0 × 10^−7^–7.5 × 10^−6^ mol L^−1^	1.9 × 10^−8^ mol L^−1^	[34]
	RNT, modified pencil graphite electrode (PGE) modified with *p*-amino benzene sulfonic acid/cucurbit(6) uril	2 × 10^−4^–1.7 × 10^−2^ mol L^−1^	1.57 × 10^−4^ mol L^−1^	[35]
	RNT, poly (chromotrope 2B) modified activated glassy carbon electrode (PCHAGCE)	1.0 ×10^−5^–4.0 ×10^−4^ mol L^−1^	5.4 ×10^−7^ mol L^−1^	[36]
	RNT, poly(thionine)-modified anodized glassy carbon electrode (PTH/GCE)	35–500 µmol L^−1^	1.5 µ mol L^−1^	[37]
	RNT, carbon paste electrode modified with the N,N-ethylene-bis(salicyllideneiminato)oxovanadium (IV) complex ((VO(salen)))	9.9 × 10^−5^–1.0 × 10^−3^ mol L^−1^	6.6 ×10^−5^ mol L^−1^	[38]
	Modified carbon paste electrode, tetraphenylborate	1.0 ×10^−6^–1.0 ×10^−2^ mol L^−1^	1.0 ×10^−6^ mol L^−1^	[39]
Proposed method	Potentiometric measurement modified RNT-PT-MgONPs and RNT-PT-Al_2_O_3_NPs sensors	1.0 × 10^−^^9^–1.0 × 10^−2^ mol L^−1^	4.9 × 10^−10^ mol L^−1^	RNT-PT-MgONPs sensor
		1.0 × 10^−10^–1.0 × 10^−2^ mol L^−1^	5.0 × 10^−11^ mol L^−1^	RNT-PT-Al_2_O_3_NPs sensor

## Data Availability

All data involved in this study are included within the text.
